# Is it reasonable to shorten the length of cemented stems? A finite element analysis and biomechanical experiment

**DOI:** 10.3389/fbioe.2023.1289985

**Published:** 2023-11-17

**Authors:** Junyan Li, Liang Xiong, Chao Lei, Xinyu Wu, Xinzhan Mao

**Affiliations:** Department of Orthopedics, The Second Xiangya Hospital, Central South University, Changsha, Hunan, China

**Keywords:** hip, joint arthroplasty, cemented stem, stem length, finite element analysis, biomechanics

## Abstract

**Background:** Uncemented short stems have been shown to optimize load distribution on the proximal femur, reducing stress shielding and preserving bone mass. However, they may adversely affect the initial stability of the stems. To date, most research conducted on short stems has predominantly centered on uncemented stems, leaving a notable dearth of investigations encompassing cemented stems. Therefore, this study aimed to investigate the length of cemented stems on the transmission of femoral load patterns and assess the initial stability of cemented short stems.

**Method:** A series of finite element models were created by gradient truncation on identical cemented stem. The impact of varying lengths of the cemented stem on both the peak stress of the femur and the stress distribution in the proximal femur (specifically Gruen zones 1 and 7) were assessed. In addition, an experimental biomechanical model for cemented short stem was established, and the initial stability was measured by evaluating the axial irreversible displacement of the stem relative to the cement.

**Result:** The maximum von-Mises stress of the femur was 58.170 MPa. Spearman correlation analysis on the shortened length and von-Mises stress of all nodes in each region showed that the *p*-values for all regions were less than 0.0001, and the correlation coefficients (r) for each region were 0.092 (Gruen Zone 1) and 0.366 (Gruen Zone 7). The result of the biomechanical experiment showed that the irreversible axial displacement of the stem relative to cement was −870 μm (SD 430 μm).

**Conclusion:** Reducing the length of a cemented stem can effectively enhance the proximal load of the femur without posing additional fracture risk. Moreover, the biomechanical experiment demonstrated favorable initial stabilities of cemented short stems.

## 1 Introduction

Total Hip Arthroplasty (THA), as one of the most successful surgical techniques of the 20th century, is the most effective treatment for various end-stage hip joint diseases. Currently, hip replacement has achieved good long-term survival rates, with an average cumulative revision rate of about 8% after 15 years for patients with osteoarthritis (Evans et al., 2019). Femoral and acetabular prostheses can be implanted using cemented or uncemented techniques. The choice of femoral fixation method is currently controversial. Supporters of uncemented fixation point out that compared to cemented femoral stems in young patients, uncemented femoral stems have good long-term survival rates (Eskelinen et al., 2006) and may be easier to revise. On the other hand, supporters of cemented fixation argue that, like elderly patients, young patients would benefit from the longer lifespan of modern cemented femoral stems (Kiran et al., 2018). Due to the unique fixation method of cemented stems, stress distribution of the proximal femur is more reasonable, with less stress shielding effect compared to uncemented stems, resulting in better preservation of bone mass and reduced long-term revision rates. Additionally, revision surgery may be easier to perform (Costi et al., 2017). Follow-up data from Australian Orthopedic Association National Joint Replacement Registry (AOANJRR) over 17 years suggested that cemented polished tapered stems had lower revision rates than commonly used uncemented stems (Babazadeh et al., 2022).

In the past decade, there has been a trend towards developing shorter femoral stems, which aims at reducing stress shielding near the femur and reducing the risk of potential proximal femoral fractures. Several uncemented short stems have shown good medium to long-term clinical results (Hossain et al., 2017; Giardina et al., 2018; Zimmerer et al., 2020). However, some researchers have reported limitations of these stems, particularly in cases of poor bone mineral density (Gruner and Heller, 2015; Shin et al., 2016). Significantly decreased bone mineral density is associated with increased risk of periprosthetic fractures when using uncemented short stems (Gkagkalis et al., 2019). Cemented short stems offer potential benefits in terms of optimizing proximal femoral loading, facilitating installation and revision procedures. Research has confirmed the good long-term survival rate of cemented short stems (Santori et al., 2019).

With the population ages, there is a significant increase in the demand for hip arthroplasty. Researchers are striving to translate the potential benefits of uncemented short stems to cemented short stems. Currently, there is limited research on cemented short stems. The purpose of this study was to investigate the influence of cemented short stem on proximal femoral load distribution and evaluate its initial stability through a combination of finite element analysis (FEA) and biomechanical experiment.

## 2 Materials and methods

### 2.1 Finite element analysis

#### 2.1.1 Finite element model

In this study, a medium-sized, left-sided, fourth-generation artificial composite femur model (3403, Sawbones, Pacific Research Laboratories, Vashon, United States) was used. To obtain the 3D model of the sawbones, it was scanned using a computed tomography (CT) machine (GE Lightspeed VCT 64, General Electric, Massachusetts, United States) with a scan interval of 1.0 mm. The CT scan data was then exported to Mimics (Version 21.0, Materialise, Leuven, Belgium) in DICOM format. After extracting the contours of each CT slice image, the contours were overlaid in three dimensions to obtain the shape and surface of the entire femur. The lines and surfaces of the 3D construction were corrected for distorted areas and then segmented to obtain the final 3D femur shape, which was exported in STL format.

The imported femur model in STL format was loaded into Geomagic Wrap (Version 2021, 3D Systems, Rock Hill, United States). The model was then remeshed to achieve a smoother surface. This involved utilizing functions such as feature removal, relaxation, and pin deletion. Additionally, the offset function was employed to create a cancellous bone model by inwardly offsetting the femur bone by 2 mm. The Mesh Doctor command was used to inspect the grid status of the mesh model. Once the inspection was passed, the precise surface interface was accessed to perform a sequence of operations. These operations included detecting and editing contour lines, constructing and repairing surface patches, constructing grids, and fitting surfaces. These actions resulted in the generation of surface models for the cortical bone and cancellous bone. Finally, the shell unit model was transformed into a solid model and exported in STEP format.

**FIGURE 1:**
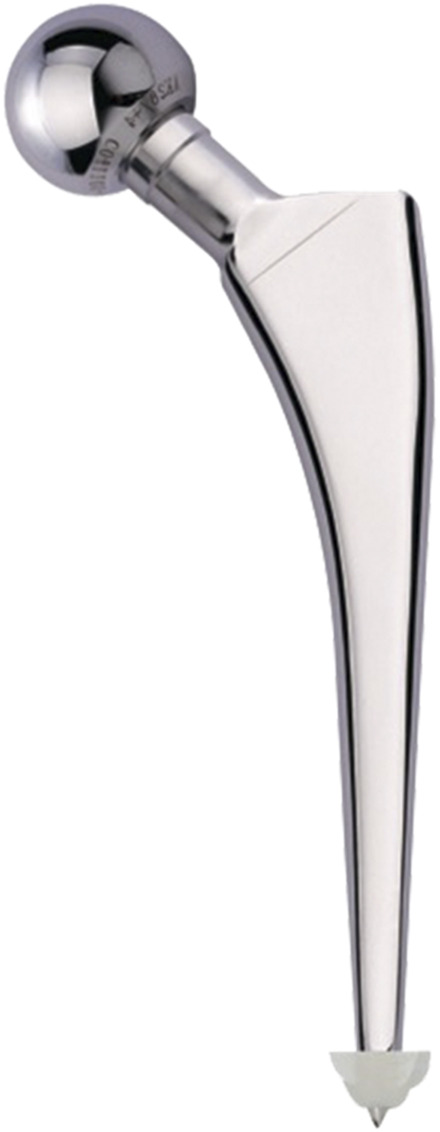
ACP stem.

We selected the ACP stem (Model: 1#, AK MEDICAL, Beijing, China) as the experimental stem. ACP stem was a high-polished, three-tapered, collarless, cemented femoral stem. The overall length of the stem was 115 mm (Figure 1). STEP format of ACP stem was collected through manufacturer datasheets.

**FIGURE 2:**
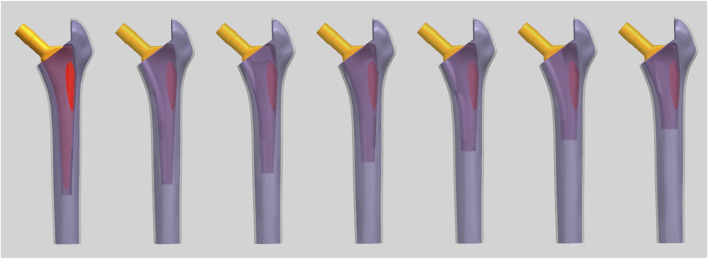
The final FEA models (From left to right, the models are labeled as ACP115 to ACP55 consecutively).

The cortical bone, cancellous bone, and stem models were imported into Unigraphics NX software (Version 12.0, Siemens, Berlin, Germany). The resection of the femoral neck was done using a standard method. The inner side of the resection was located 10 mm above the lesser trochanter horizontally, while the outer side was located at the level of the base of the femoral neck. The femoral neck was cut at a 45° angle along the line connecting these two points in the coronal plane, and the femoral head was subsequently removed. To minimize computational load, the distal femur was removed. Every 10 mm of the distal end of the stem was horizontally truncated once, for a total of six truncations, resulting in seven sets of stems with lengths ranging from 55 mm to 115 mm. They were named ACP55 to ACP115 based on the stem length. The length of ACP75 was equal to twice the vertical distance from the highest point of the greater trochanter of the femur to the lowest point of the lesser trochanter. So ACP55 to ACP75 were regarded as short stems by Feyen and Shimmin. (2014). The longitudinal axis of the stem was parallel to the anatomical axis of the femur in both the coronal and sagittal planes. The stem was assembled in the femur with a anteversion of 15°, and the height of the rotation center of the femoral head aligned with the highest point on the greater trochanter horizontally. By using the intersection function, the intersection surface between the stem and cancellous bone was obtained. This surface was then offset outward by 2 mm to obtain a 2 mm-thick cement mantle. The cortical bone region was removed by Boolean operations to account for the space occupied by cancellous bone and the stem. The stem-occupied space was removed from the cancellous bone. Finally, seven sets of final models were obtained and exported in PRT format as shown in Figure 2.

#### 2.1.2 Material properties and meshing

We assumed that the bone structure had homogeneous and isotropic linear properties. The material properties were obtained from the manufacturer, respectively for cortical bone (E = 16.7 GPa, *ν* = 0.3), trabecular bone (E = 0.155 GPa, v = 0.3), bone cement (E = 2.2 GPa, *ν* = 0.3), and ACP stem (E = 210 GPa, *ν* = 0.3) (Naghavi et al., 2023).

We partitioned all finite element models into tetrahedral 10-node meshes. To assess mesh convergence, we constructed six groups of finite element models with different mesh densities and performed finite element analysis with the same boundary conditions. The peak value of the von-Mises stress in the femur was considered the convergence observation indicator, defined as the numerical difference between the two successive solutions under the same loading conditions being less than 5% to determine mesh convergence. Based on the results of the mesh convergence analysis, the optimal mesh sizes were determined to be: 2 mm for cortical bone, 2 mm for cancellous bone, 1 mm for bone cement, and 1.5 mm for the stem. The result of mesh quality evaluation showed that the average values of jacobian ratio and skewness were 1.037 and 0.252 (Ruggiero et al., 2019).

#### 2.1.3 Loading and boundary conditions

Finite element analysis was performed using Ansys workbench (Version 2021 R1, ANSYS Inc, Canonsburg, United States). The ASTM F2996–13 and ISO 7206–4:2010(E) were taken into consideration when determining the loading and boundary conditions (Bergmann et al., 2016).A load of 2300N was applied to the point at the center of the maximum offset femoral head, at a 12° angle to the anatomical axis of the femur. The distal end of the femur was set as the fixed support surface. All the contact areas were considered bonded except the cement-stem interface. The coefficient of friction 0.25 was considered between cement and stem interface (Verdonschot and Huiskes, 1996).

#### 2.1.4 Observation indicators and statistical analysis

**FIGURE 3:**
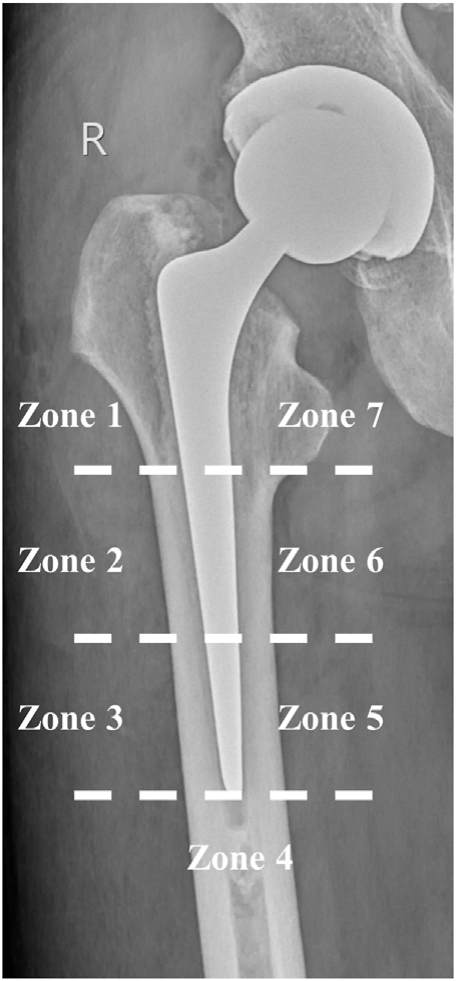
Gruen zones in cemented stems.

The main purpose of this finite element analysis was to observe the following factors and statistical methods in different lengths of cemented stems after implantation in the femur: (1) Peak values and distribution characteristics of von-Mises stress in the femur; (2) Characteristics of the distribution of nodal von-Mises stress in the proximal femur. We chose Gruen zones 1 (number of nodes: 755) and 7 (number of nodes: 755) as the regions of interest (Figure 3) (Gruen et al., 1979). Because these areas are less affected by the design of the femoral stem (de Waard et al., 2021). Statical analysis was performed using IBM SPSS (Version 23, IBM, Armonk, United States). The normality test indicated that the data was nonparametric, hence the summary statistics were represented by the median and interquartile range (IQR). Spearman correlation analysis was used to find the relationship between the length of the stem and von-Mises stress in the proximal femur. When *p* < 0.05, it was considered to indicate correlation. In order to ensure comparability of the results, the Gruen partitioning of the ACP115 group was used as the partitioning criterion for the femoral models in each group.

### 2.2 Biomechanical experiments of cemented short stem

We used a stem with a length at the junction of the standard stem and the short stem as the experimental object, which was referred to as the ACP75 stem mentioned in the above text. The distal part of the stem was cut using a low-speed water-cooled saw to minimize damage to the remaining part of the prosthesis. After the cutting was completed, the cut surface was rounded and polished to reduce local stress concentration and adverse effects on cement during subsequent implantation.

Implant structures were prepared by experienced orthopedic surgeon (X.M). A standard femoral neck cut was made approximately 1 cm near the lesser trochanter, and then a suitable broaching rasp of the stem size was used to broach the bone for stem placement.

A distal cement plug was introduced at the distal end located at the tip of the stem. Then, Simplex bone cement (Stryker Orthopedics, Mahwah, United States) was used to bond the stem to Sawbones using a stem centralizer. A total of six models were prepared for the following experiments.

**FIGURE 4:**
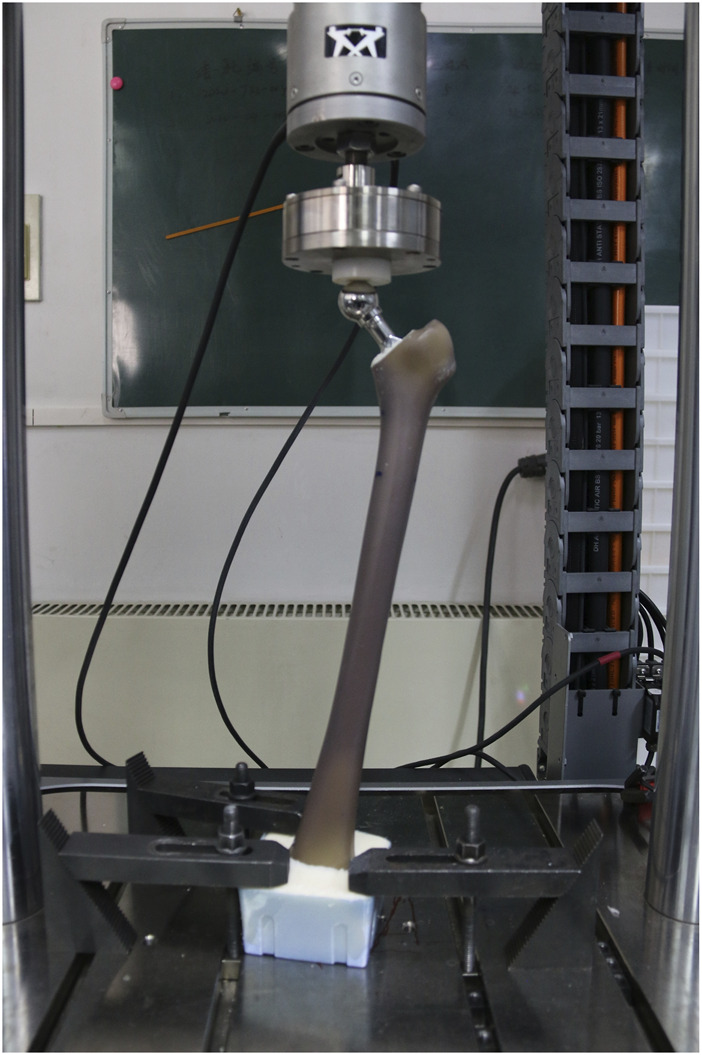
The testing machine. The femur and stem are fitted in the Instron.

The femoral models were installed in the material testing machine (ElectroPuls E10000, Instron, Norwood, United States) and subjected to vertical loading. To ensure that the load was introduced without creating any moments, a ball bearing was placed between the device and the load cell (Figure 4). The material testing machine applied 100,000 dynamic sinusoidal load cycles at a frequency of 2 Hz between 100 and 1600 N to simulate the load during the first 6 weeks *in vivo* condition (Freitag et al., 2021). The axial displacement between the stem and the cement mantle was used as an observation parameter to evaluate the initial stability of the stem. The measurement process was performed using the PLMLAB DIC-3D system (PLMLAB Sensor Tech, Nanjing, China). This system was based on the principle of binocular stereo vision and uses three-dimensional digital image correlation methods to measure the three-dimensional shape and the three-dimensional strain field of the tested object’s surface under loading. The axial displacement of the stem was determined by measuring the difference in axial distance between the highest point of the stem shoulder and the highest point of the cement mantle in the coronal plane.

## 3 Results

### 3.1 Peak values and distribution characteristics of femoral von-Mises stress

**FIGURE 5:**
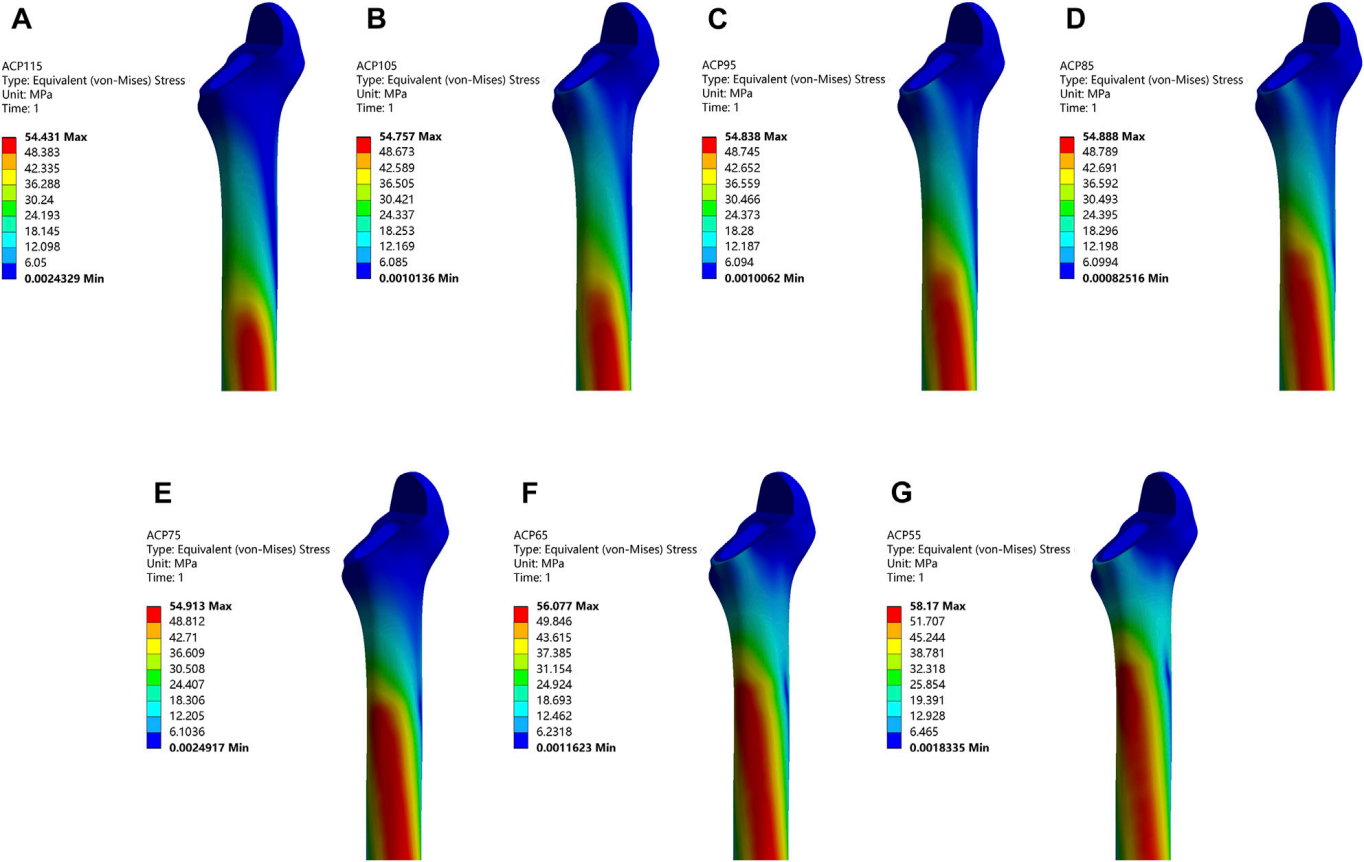
Femoral von-Mises stress distribution under different stem lengths.

Total seven models with different stem length were analyzed, as shown in Figure 5. In general, the peak von-Mises stress tended to increase with a decrease in the stem length. Furthermore, the location of the peak Von-Mises stress was gradually shifted from the medial distal femur to the medial proximal femur. ACP115 group was 54.431 MPa, ACP105 group was 54.757 MPa, ACP95 group was 54.838 MPa, ACP85 group was 54.888 MPa, ACP75 group was 54.913 MPa, ACP65 group was 56.077 MPa, ACP55 group was 58.170 MPa. The rates of increase in peak Von-Mises stress, compared to the ACP115 group, were as follows: 0.599% (ACP105), 0.748% (ACP95), 0.840% (ACP85), 0.886% (ACP75), 3.024% (ACP65), and 6.869% (ACP55).

### 3.2 Distribution of von-Mises stress in the proximal femur

**FIGURE 6:**
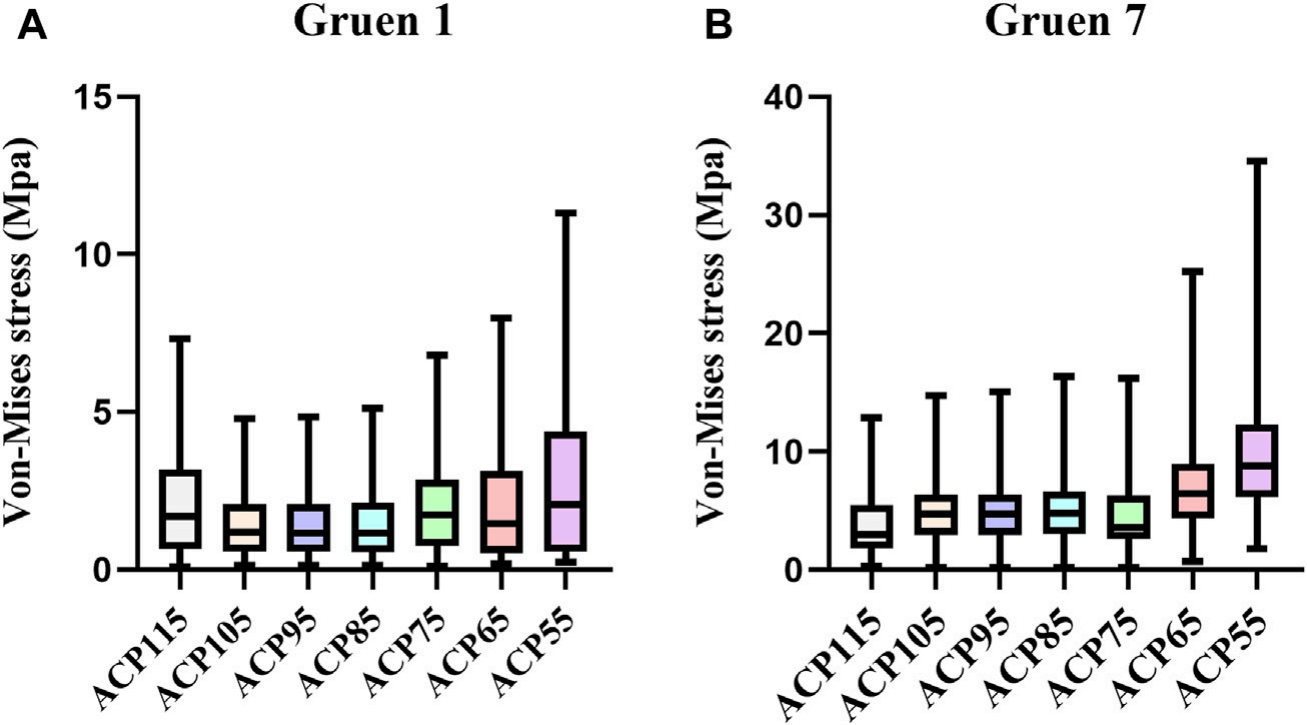
Nodal von-Mises stress in the proximal femur under different stem lengths. **(A)** Gruen zone 1 **(B)** Gruen zone 7.

The nodal von-Mises stress in the proximal femur of each model was calculated from the finite element results and plotted in Figure 6. In Gruen zone 1, the von-Mises stress for each group model was as follows: ACP115 (1.682 MPa, IQR 0.651–3.173), ACP105 (1.177 MPa, IQR 0.574–2.079), ACP95 (1.166 MPa, IQR 0.567–2.063), ACP85 (1.173 MPa, IQR 0.555–2.12), ACP75 (1.749 MPa, IQR 0.738–2.857), ACP65 (1.467 MPa, IQR 0.530–3.116), ACP55 (2.071 MPa, IQR 0.575–4.368). In Gruen zone 7, the von-Mises stress for each group model was as follows: ACP115 (2.987 MPa, IQR 1.810–5.522), ACP105 (4.737 MPa, IQR 2.973–6.385), ACP95 (4.711 MPa, IQR 2.961–6.395), ACP85 (4.817 MPa, IQR 3.066–6.617), ACP75 (3.597 MPa, IQR 2.639–6.276), ACP65 (6.481 MPa, IQR 4.378–8.953), ACP55 (8.823 MPa, IQR 6.172–12.332).

**FIGURE 7:**
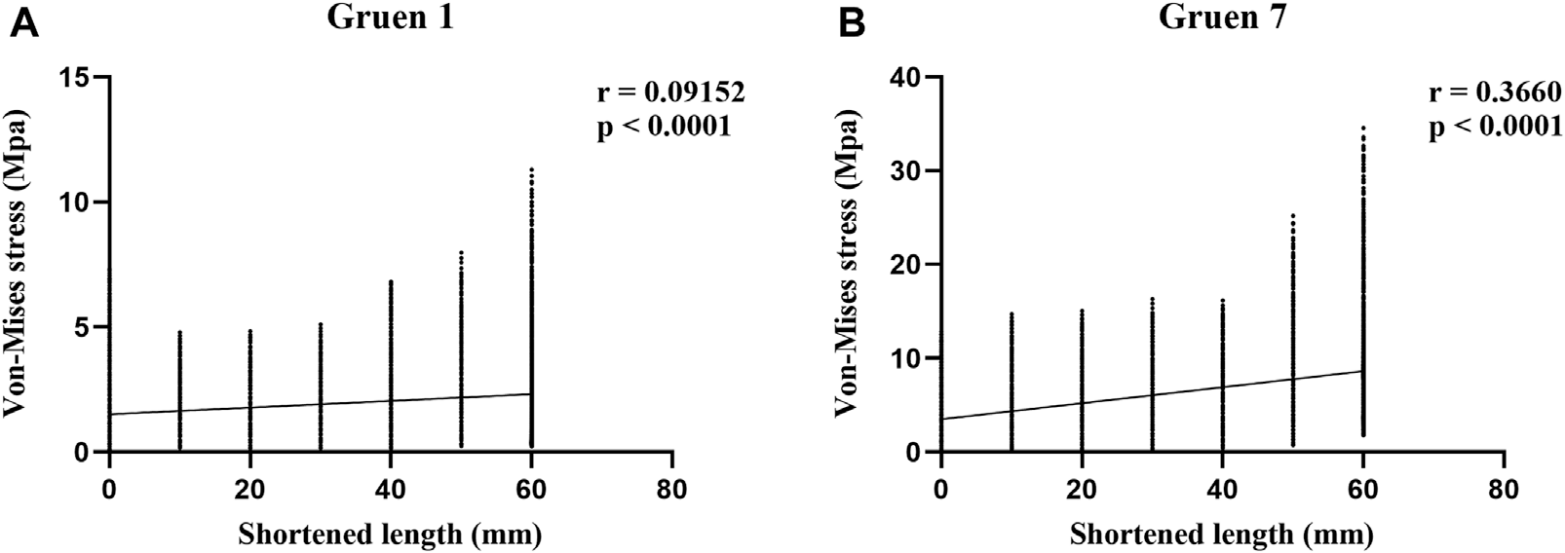
Results of Spearman correlation analysis between shortened length and von-Mises stress in the proximal femur. **(A)** Gruen zone 1 **(B)** Gruen zone 7.

In order to investigate the correlation between von-Mises stress in different regions of the femur and the length of the stem, Spearman correlation analysis was performed on the von-Mises stress and shortened length of all nodes in each region, as shown in Figure 7. The results showed that the *p*-values for all regions were less than 0.0001, and the correlation coefficients (r) for each region were 0.092 (Gruen Zone 1) and 0.366 (Gruen Zone 7), which indicating a positive correlation between von-Mises stress in the proximal femur and the shortened length of the stem.

### 3.3 Primary stability evaluation

After 100,000 loading cycles, the irreversible axial displacement of the ACP75 stem relative to bone cement was −870 μm (SD 430 μm).

## 4 Discussion

Currently, limited research exists on cemented short stems. This study investigated the impact of cemented stem length on femoral load and the initial stability of cemented short stems. To eliminate the influence of metaphyseal design factors on experimental results, we progressively truncated the same stem. This approach allowed us to clearly comprehend the trend of changes in various observed indicators as the length of the cemented stem changed. Our finite element analysis demonstrated that shortening the length of cemented stems effectively enhanced the proximal load of the femur, while having minimal effects on the femur’s peak stress. The peak von-Mises stresses in all groups were far below the ultimate strength of 133 Mpa, which did not increase the risk of fracture. Additionally, we conducted fatigue testing to simulate the load conditions experienced by cemented short stems during the first 6 weeks *in vivo*. Based on our findings, we observed an irreversible axial displacement of −870 μm for the cemented short stem, which fell below the predetermined criterion of 5 mm for prosthesis loosening (Santori et al., 2019). This result provided evidence for the favorable initial stability of cemented short stems.

This study demonstrated results similar to those of uncemented stems. Bieger et al. and Arno et al. have argued that reducing the stem length can decrease proximal stress shielding without compromising initial stability (Arno et al., 2012; Bieger et al., 2012). In a comparison between the Alloclassic hip system and the Mayo short stem, Boyle et al. discovered that the Mayo stem was more effective in load transmission to cancellous bone and reducing proximal bone loss (Boyle and Kim, 2011). Østbyhaug et al. conducted research on the ABG-1 stem and determined that shortening the stem by 40–50 mm could transmission stress more effectively in the metaphysis and diaphysis (Østbyhaug et al., 2009). However, shortening the uncemented stem may impact initial stability. Van Rietbergen et al. found that shorter stems had higher shear stress near the distal lateral side compared to standard stems, which could potentially lead to decreased initial stability (van Rietbergen and Huiskes, 2001). Ong et al. compared longer and shorter versions of the Omnifit hydroxyapatite stem and observed that although the shorter design had greater potential for bone formation on the medial side, the displacement of the bone-prosthesis interface at the tip of the short stem was 40%–94% greater than that of the longer stem, which may result in patient discomfort (Ong et al., 2009). Cement fixation had a significant impact on load transfer at the proximal femur (Scheerlinck and Casteleyn, 2006). The study by Freitag et al. found that cemented Optimys short stem demonstrated a smaller irreversible axial displacement after fatigue testing compared to cemented conventional straight twinSys stem (−20.4 µm ± 38.3 µm/−61.4 µm ± 92.8 µm) (Freitag et al., 2021). Although no statistical differences were observed in the result. Thomsen et al. compared the maximum fracture load and fracture pattern of cemented and uncemented stems in non-osteoporotic bone and discovered that the maximum fracture load of the cemented stem was significantly higher (Thomsen et al., 2008). Similarly, Klasan et al. conducted an experiment on cadaver bones comparing the failure loads of cemented and uncemented stems. They found that the failure load of the cemented stem increased by 25% compared to the latter (Klasan et al., 2019). The utilization of cement technology may offer a solution to the low initial stability observed in uncemented short stems.

In recent years, several clinical studies have been published regarding cemented short stems. A study based on AOANJRR compared the 7-year follow-up results of the short and standard Exeter stem. Despite the short stem being used in a larger proportion of potential difficult cases with developmental dysplasia of the hip, there was no significant difference observed in the cumulative revision rate between the short and standard Exeter stem (Choy et al., 2013). However, another study based on the New Zealand National Joint Registry produced different result. This study found that the revision rate of the standard Exeter stem was significantly lower than that of the short Exeter stem with an offset of 35.5 mm. On the other hand, the revision rate of the shorter Exeter stem with an offset of 37 mm was similar to that of the standard stem (Wyatt et al., 2020). Another recent study also indicated that femoral stem with smaller offset carried a higher risk of revision (Wyatt et al., 2019). Therefore, for the Exeter stem, offset appears to be more important than stem length, as the proximal part provides rotational stability (Wilson et al., 2012). In addition to revision rates, a randomized controlled trial has compared the functional outcomes between short and standard Exeter stems in total hip arthroplasty. The result of this trial showed that, at an average of 2 years postoperatively, the short Exeter stem exhibited similar hip joint function, health-related quality of life, and patient satisfaction compared to the standard stem (Gaston et al., 2023). Although a greater rate of varus malalignment was found in short stem group, which may affect the stem survival rate in the future.

However, the short Exeter stem was not a truly representative short stem in the conventional sense. It was designed specifically for patients with smaller femurs. The longest follow-up results for cemented short stems were derived from the study conducted by Santori et al. The results showed that the survival rate of the Friendly cemented short stem, with aseptic loosening as the endpoint, was 100% with a maximum follow-up of 11.2 years (Santori et al., 2019). However, it was important to note that the Friendly short stem was only a modification of the Exeter stem concept, with a reduced length, limiting its comparability to the novel generation of calcar-guided short-stem concept. To date, there have been no new-generation cemented short stems used clinically. The utilization of the line-to-line technique in the development of cemented short stems may present a promising alternative for the treatment of osteoporotic bone conditions (Azari et al., 2021).

There are some limitations in this study that should be acknowledged. Firstly, the finite element analysis was performed under static load conditions, which only provided limited results. It was important to consider more realistic load conditions, such as walking, climbing stairs, and running (Kwak et al., 2021). Previous research has demonstrated that the torque generated during stair climbing poses the greatest risk for cement failure (Bergmann et al., 1995). However, in dynamic situations, it is crucial to understand the interplay between the femoral and the acetabular prosthesis. Recently, scholars have developed a novel 3D contact-lubrication model to calculate the wear performance of hip prostheses during the gait process, and they have achieved encouraging results. This may assist us in better simulating the kinematic characteristics after hip arthroplasty (Ruggiero and Sicilia, 2020). Secondly, we were hindered by the lack of a femoral canal rasp specifically designed for the shortened stem. Consequently, we utilized a femoral canal rasp that corresponded to the pre-cut stem, leading to a longer cement mantle at the distal end. However, the mechanical strength of the Sawbones primarily derived from the cortical bone, which remained unaffected by this issue. Therefore, we believed that the impact on the result was minimal. Lastly, the simulated loading employed during the first 6 weeks could only capture the initial characteristics following the implantation of the cemented short stem. The medium and long-term characteristics can only be obtained by conducting long-term clinical follow-up studies *in vivo*.

## 5 Conclusion

The findings of the finite element analysis indicated that, similar to an uncemented stem, reducing the length of a cement stem could effectively enhance the proximal load in the femur without posing additional fracture risk. Moreover, the biomechanical experiment validated the favorable initial stabilities of cemented short stems. However, further investigations are required to ascertain whether these findings can translate into clinical benefits.

## Data Availability

The raw data supporting the conclusion of this article will be made available by the authors, without undue reservation.
